# Supplementary material for: Cortical recurrence supports resilience to sensory variance in the primary visual cortex
Source: Commun Biol. 2023 Jun 23;6:667. doi: 10.1038/s42003-023-05042-3 (PMC10290066; doi:10.1038/s42003-023-05042-3)
Supplement: Supplementary file 2 — Supplementary Information [file 42003_2023_5042_MOESM2_ESM.pdf]

Supplementary information

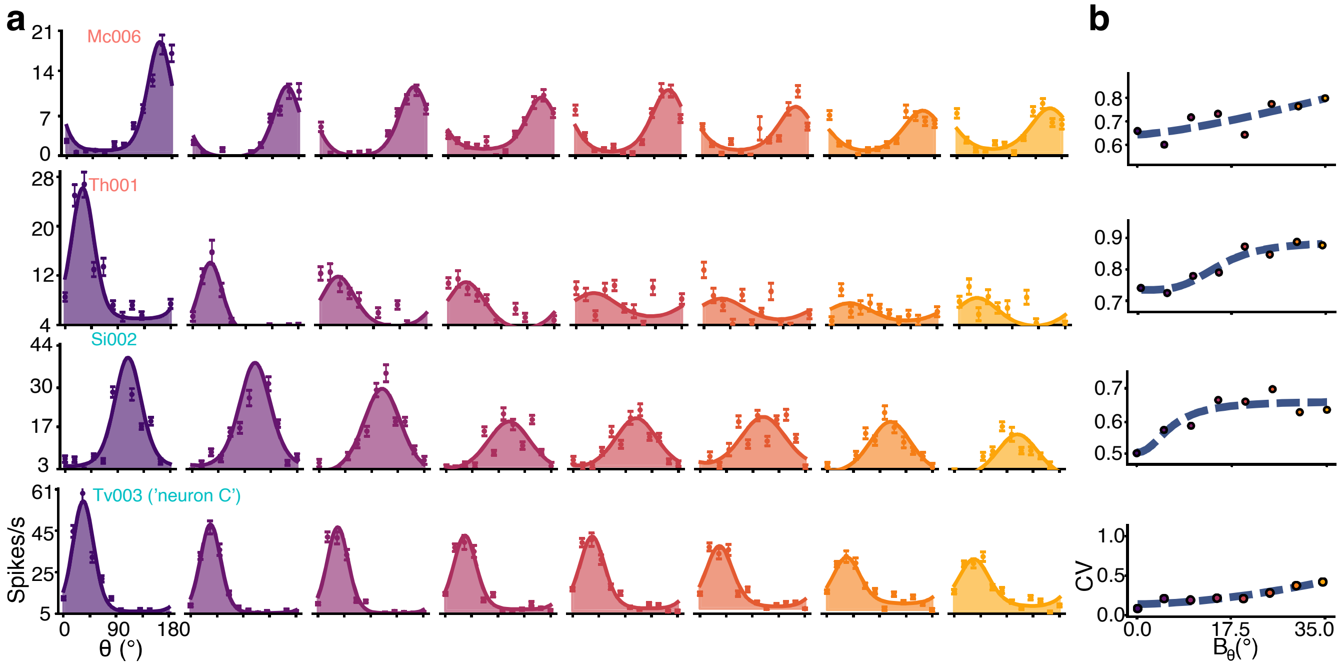

Supplementary Figure 1. Additional examples of single neuron tuning curves and VTF. (**a**) Tuning curves of four more neurons, stimulated with Motion Clouds of increasing variance (controlled by B_θ) from left to right. Colored dots represent the mean firing rate across trials (baseline subtracted, 300 ms average), error bar the standard error and solid lines a fitted von Mises function. (**b**) Variance-tuning functions (VTF), measuring the changes of orientation tuning measured by the circular variance (CV, colored dots) as a function of stimuli variance, fitted with a Naka-Rushton (NKR) function (dashed curves, parameters shown in light gray).

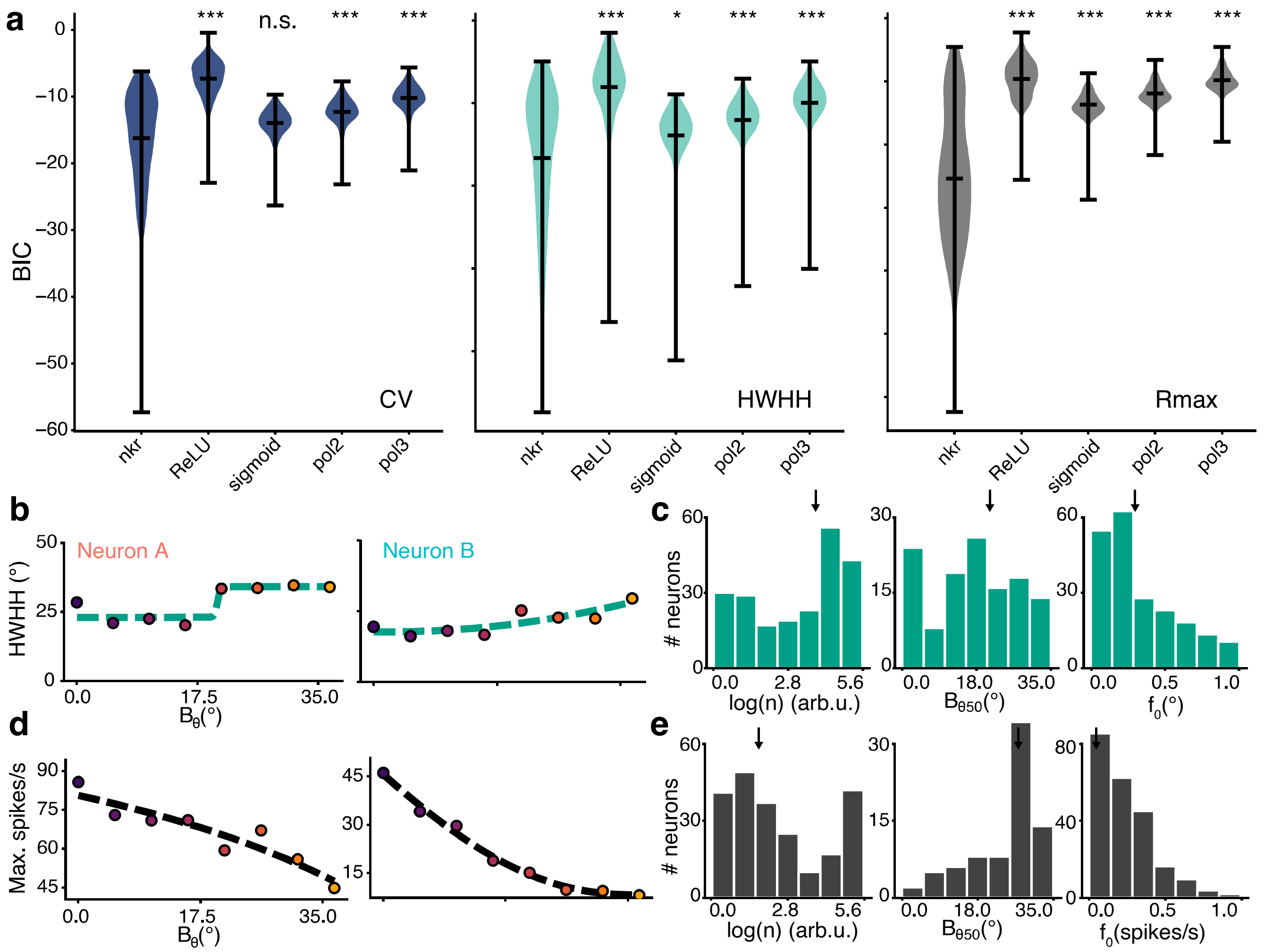

Supplementary Figure 2. Naka-Rushton function fitting of VTFs. (**a**) Violin plot of the Bayesian Information Criterion (BIC) of the CV curves of all recorded neurons. Each violin plot represents a different type of fitted equation, respectively: Naka-Rushton (`nkr`, see Methods) ; Rectified Linear Unit (`ReLU`, $f(x) = max(0,x)$) ; logistic function (`sigmoid`, $f(x) = \frac{e^x}{e^x+1}$) ; second degree polynomial function (`pol2`, $f(x) = ax^2 + bx + c$) and third degree polynomial function (`pol3`, $f(x) = ax^3 + bx^2 + cx + d$). A lower BIC indicates less information lost in the fitting process, hence a better fitting model. Naka-Rushton curves were chosen over sigmoid functions for the skewness of the BIC distributions towards negative values, as well as the explainability of their parameters. `n.s.`, not significant; $\star$, p < 0.05; $\star\star$, p < 0.01; $\star\star\star$, p < 0.001 (Kruskal-Wallis H-test, post-hoc Dunn Pairwise test, Bonferroni corrected) (**b**) Variance-HWHH function, fitted with a Naka-Rushton function. (**c**) Histograms of the NKR parameters (in the [5%;95%] range of possible NKR fitting values) for the 249 recorded units. Median values are indicated by a black arrow ($\log(n) = 3.8$, $B_{\theta 50} = 16.5°$, $f_0 = 0.25$). Parameter f_0 is given in normalized HWHH values. (**d**) Same as (b), with maximum firing rate as a function of input variance. (**e**) Same as (c), for maximum firing rate. Median values are indicated by a black arrow ($\log(n) = 2.15$, $B_{\theta 50} = 28.3°$, $f_0 = 0.09$). Parameter f_0 is given in normalized firing rate values

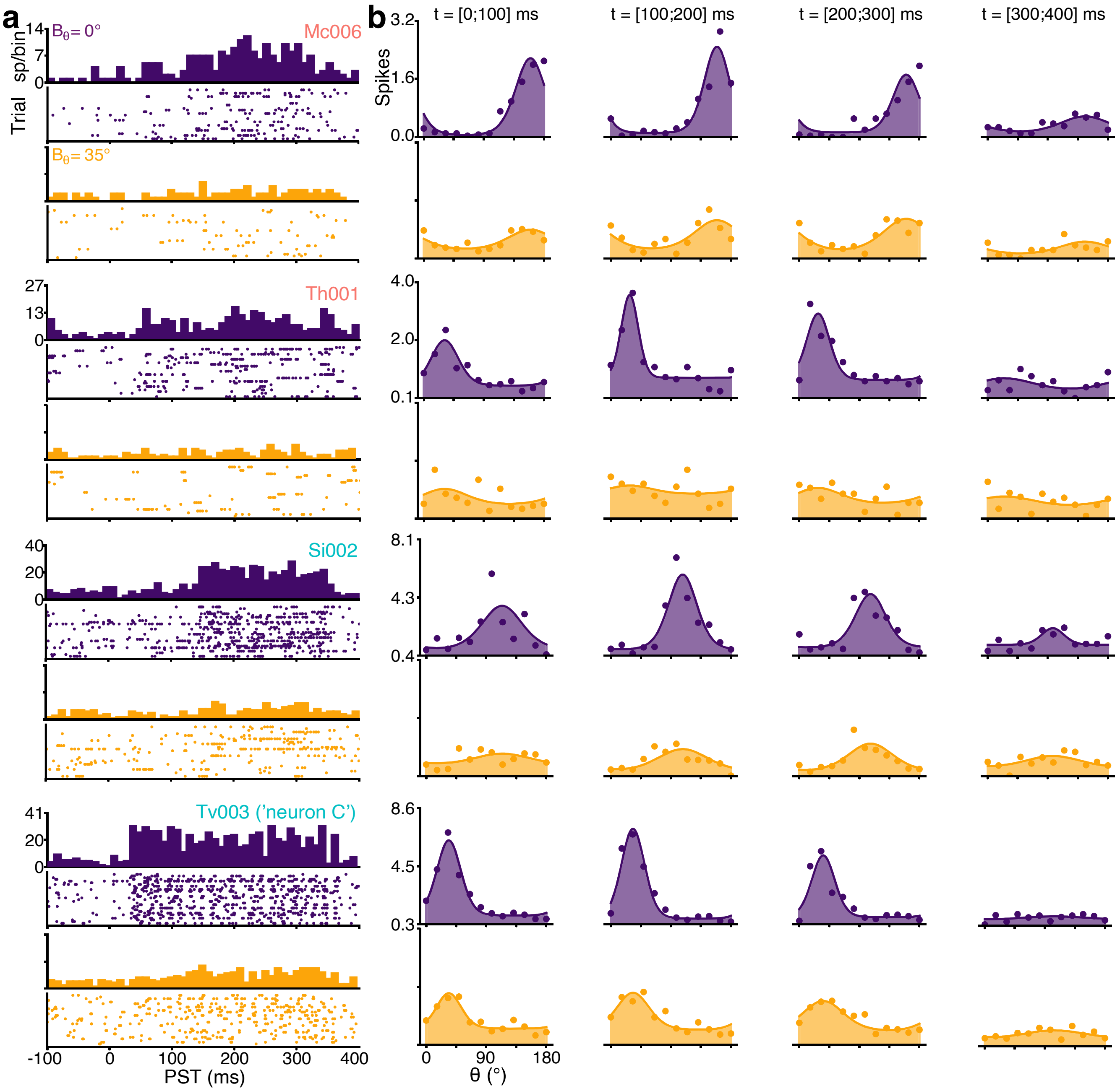

Supplementary Figure 3. Additional examples of dynamical properties of neurons. (**a**) Peristimulus time (PST) histogram and associated rasterplot of the four additional example neurons, for Motion Clouds with lowest ($B_\theta = 0°$, purple) and highest ($B_\theta = 35°$, yellow) variance. (**b**) Dynamics of the tuning curves shown in Figure 2 in 100 ms windows, starting at the time labelled atop of each column..

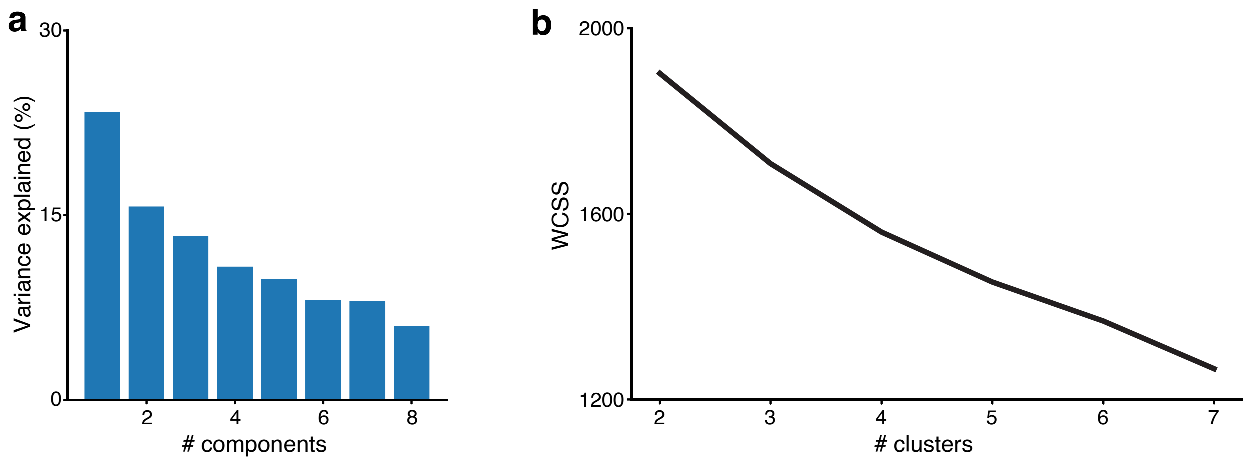

Supplementary Figure 4. Graphical reports of clustering analysis. (**a**) Fraction of variance explained as a function of the number of components used in the Principal Component Analysis. (**b**) Within-Cluster-Sum-of-Squares (WCSS) of the K-means clustering, expressed as a function of the number of clusters. An empirical way to select the number of clusters is to typically take the "elbow" of such a curve, if existent.

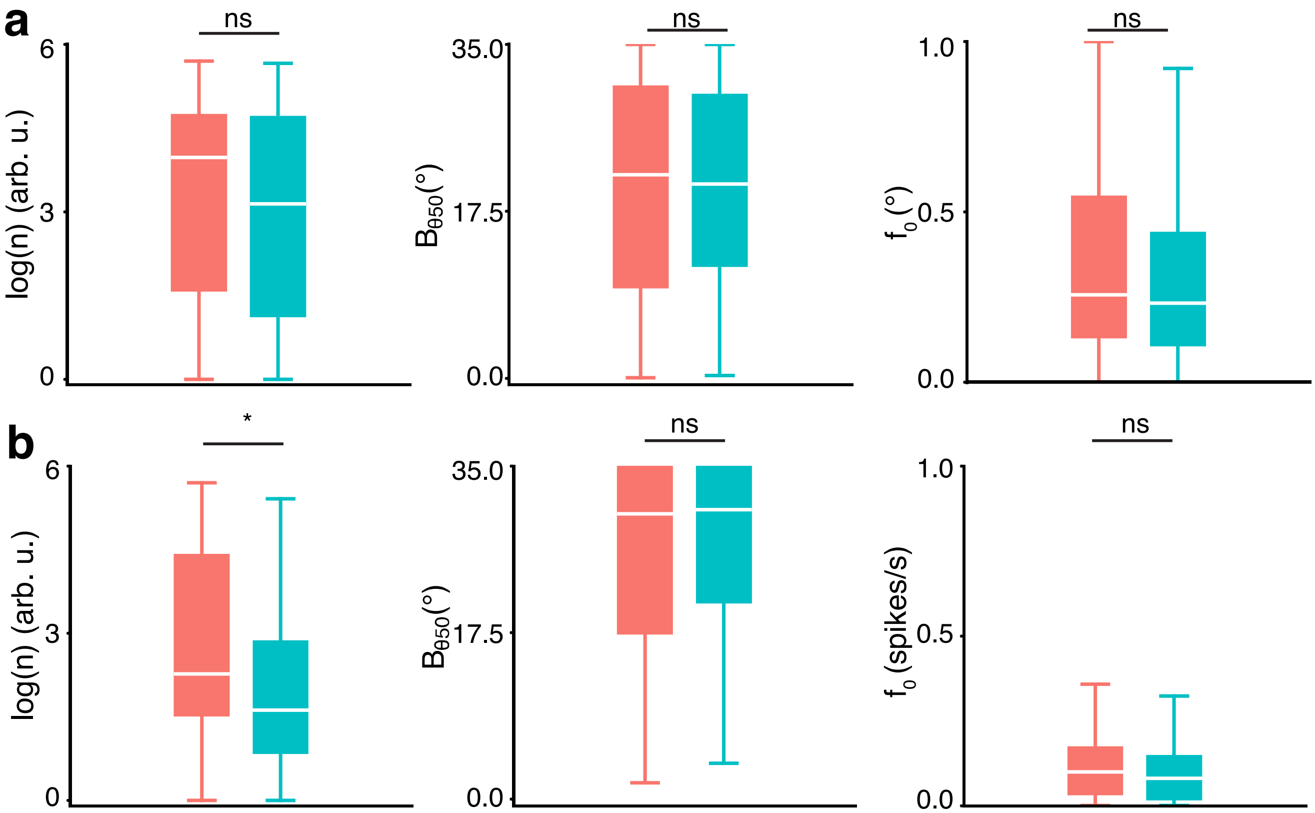

Supplementary Figure 5. Additional clustering variables. (**a**) Boxplot of the VTF parameters for HWHH functions. (**b**) Boxplot of the VTF parameters for maximum response functions.

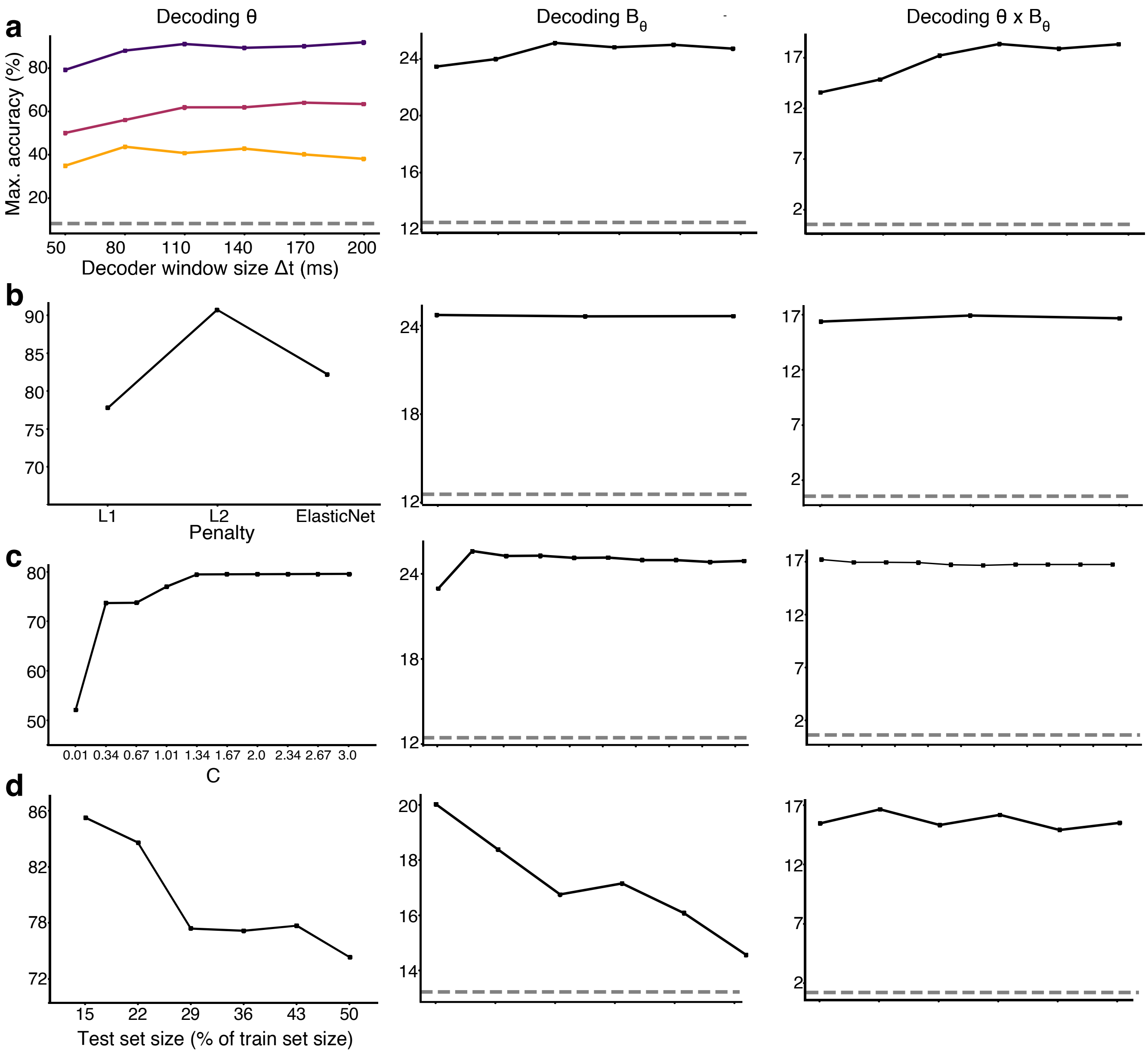

Supplementary Figure 6. Parameters evaluation of the decoders. (**a**) Optimization of the length of the time window ΔT, which controls the length of the integration time of the decoder (see Methods), for orientation decoders (left column), variance decoders (middle column) and orientation x variance decoders (right column). (**b**) Optimization of the penalization norm applied to the decoder, which controls the metric by which the classification error is minimized. l_1 and l_2 are defined as $\sum_i |x_i|$ and $\sqrt{\sum_i |x_i|^2}$, where x is a set of features fed to the classifier. The penalty norm ElasticNet linearly combines these two norms, here with equal weighting applied to both norms. (**c**) Optimization of the parameter C controls the regularization strength, which measure the magnitude of the penalty applied to large parameters, in order to prevent overfitting on reduced sets of data. (**d**) Optimization of the percentage of data kept out of the training set to evaluate the decoder's accuracy.

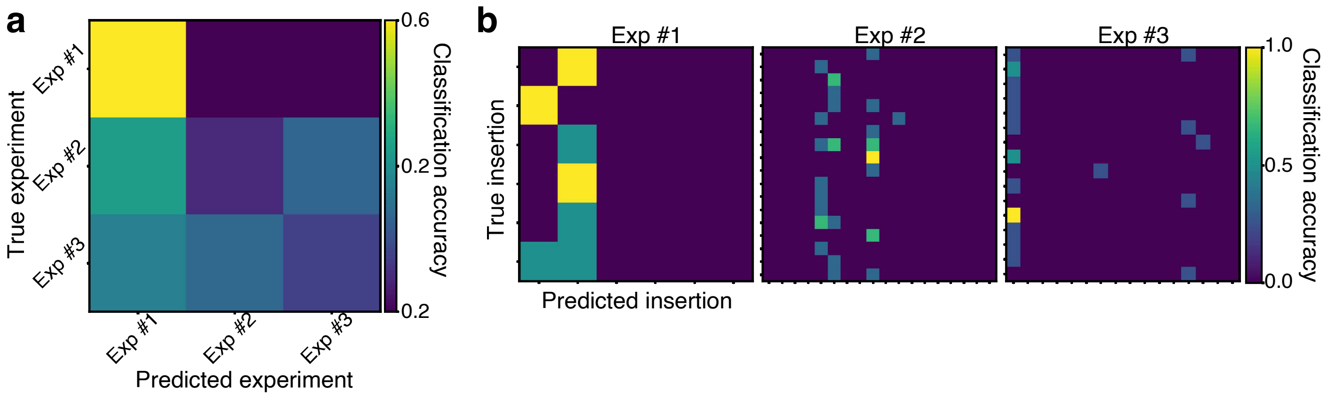

Supplementary Figure 7. Decoding the experiment's identity (the specific experiment which yielded the spikes) or insertion's identity (the specific insertion which yielded the spikes) of neurons does not produce any significant result, thereby validating the fusion of multiple datasets in the decoding process. (**a**) Confusion matrix of a decoder trained to retrieve the experiment identity using three groups of 30 neurons (bootstrapped 1000 times). (**b**) Confusion matrices of three decoders trained to retrieve the insertion identity of the neurons recorded in each individual experiment.

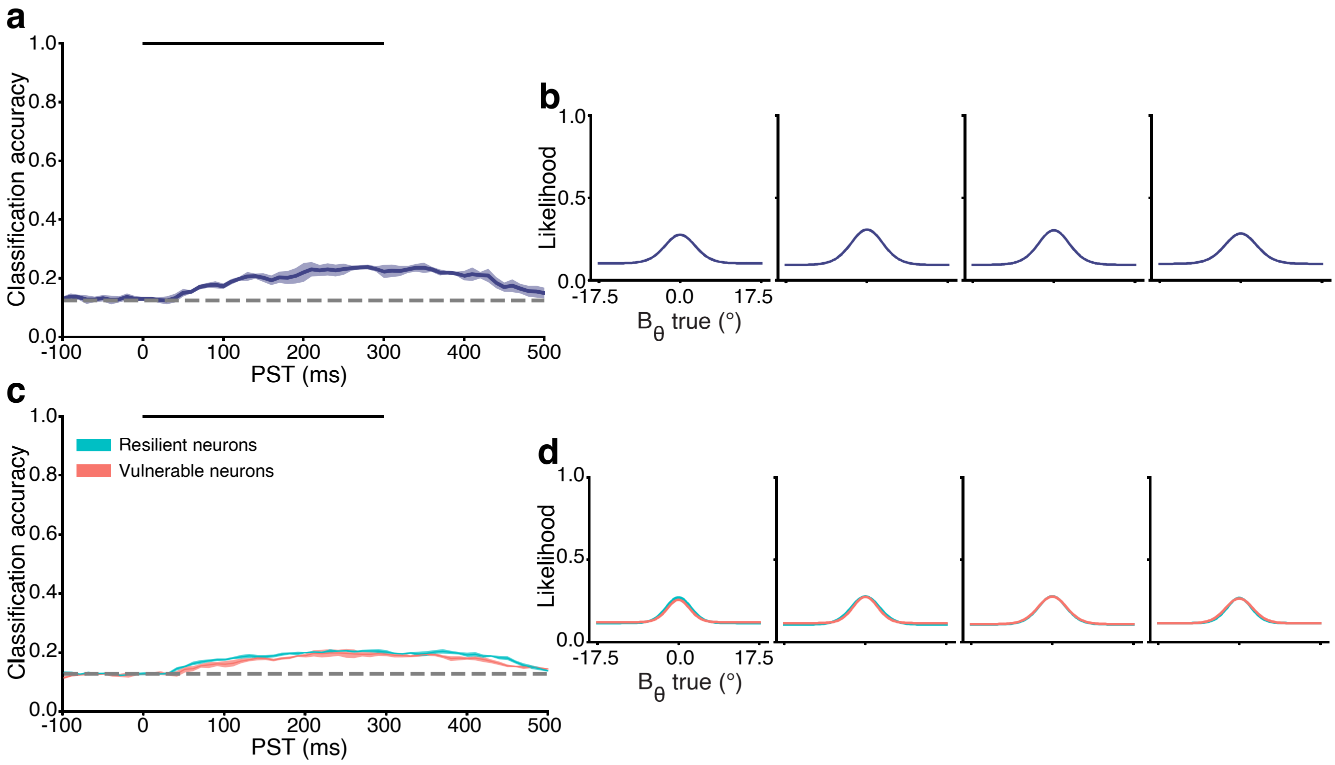

Supplementary Figure 8. Orientation variance cannot be accurately decoded from the population activity. (**a**) Time course of a decoder trained to retrieve the variance B_θ of Motion Clouds. Solid dark line represents the mean accuracy of a 5-fold cross validation and filled contour the SD. Decoding at chance level (here, 1/8) is represented by a gray dashed line. (**b**) Population tuning curve of the decoder, representing the likelihood of decoding as a function of error on variance. (**c**) Same as Supplementary Figure8a, trained with spikes from either resilient or vulnerable neurons. (**d**) Same as Supplementary Figure8b, trained with spikes from either resilient or vulnerable neurons.

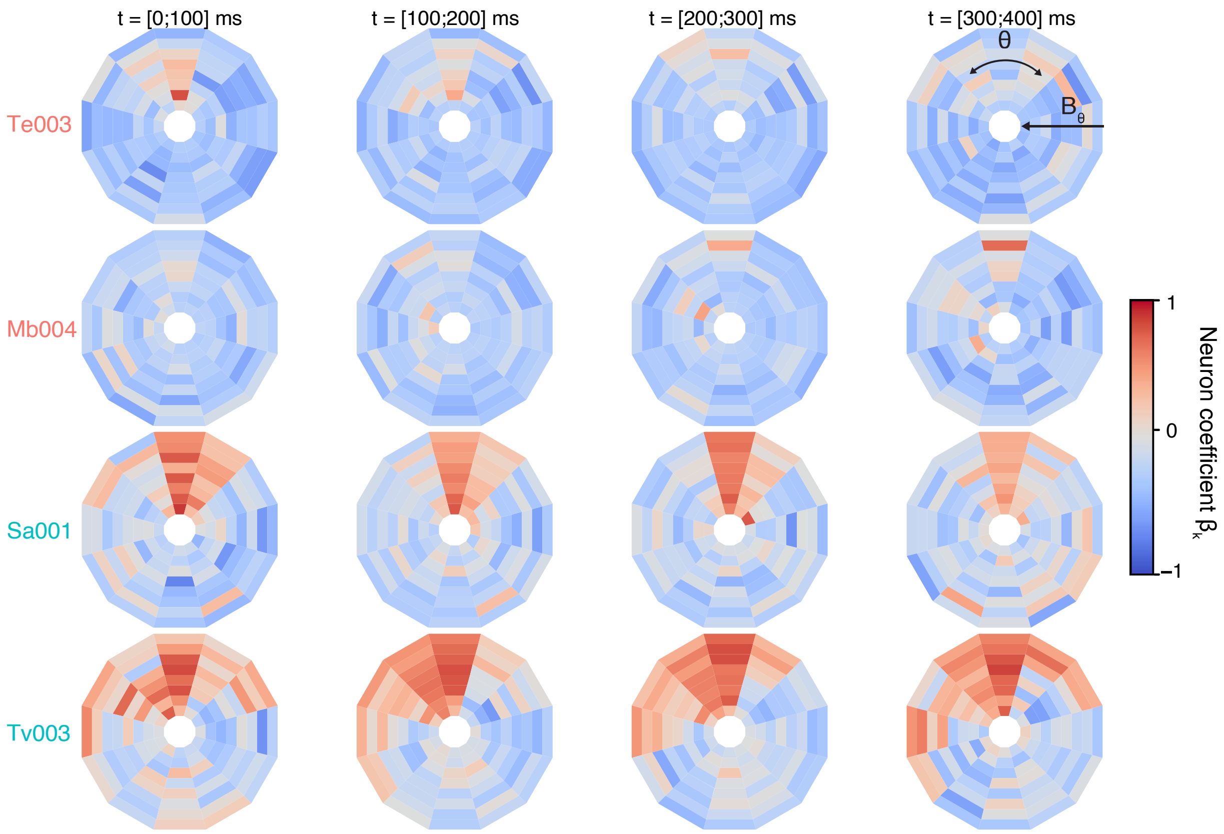

Supplementary Figure 9. Polar plot of coefficient matrices from two single vulnerable neurons (top rows) and two single resilient neurons (bottom rows). As in Figure 6e, the angle of each bin represents the error on the θ identity of the stimulus, Δ_θ, and the eccentricity corresponds to the coefficient for each B_θ (highest variance at the center). The temporal evolution of the coefficients from the decoder is normalized for each neuron.
